# Discovery of a New Anæsthetic Agent More Efficient Than Sulphuric Ether

**Published:** 1848-01

**Authors:** J. Y. Simpson

**Affiliations:** Professor of Midwifery in the University of Edinburgh, Physician-Accoucheur to her Majesty in Scotland, &c.


*Discovery of a New Anæsthetic Agent more efficient than Sulphuric Ether.* By J. Y. Simpson, M. D., Professor of Midwifery in the University of Edinburgh, Physician-Accoucheur to her Majesty in Scotland, &c.—At the first winter meeting of the Medico-Chirurgical Society of Edinburgh, held on the 10th November last, I had an opportunity of directing the attention of the members to a new agent which I had been using for some time previously for the purpose of producing insensibility to pain in surgical and obstetric practice.

This new anæsthetic agent is chloroform, chloroformyle, or perchloride of formyle.* Its composition is expressed by the chemical formula $C^2$ H $Cl^3$. It can be procured by various processes, as by making milk of lime or an aqueous solution of caustic alkali act upon chloral; by distilling alcohol, pyroxilic spirit, or acetone with chloride of lime; by leading a stream of chlorine gas into a solution of caustic potass in spirit of wine, &c. The resulting chloroform obtained by these processes is a heavy, clear, transparent liquid, with a specific gravity as high as 1·480. It is not inflammable. It evaporates readily, and boils at 141°. It possesses an agreeable, fragrant, fruit-like odour, and a saccharine, pleasant taste.

As an inhaled anæsthetic agent, it possesses, I believe, all the advantages of sulphuric ether, without its principal disadvantages.

1. A greatly less quantity of chloroform than of ether is requisite to produce the anæsthetic effect, usually from a hundred to a hundred and twenty drops of chloroform being sufficient, and with some patients much less. I have seen a strong person rendered completely insensible by seven inspirations of thirty drops only of the liquid.

2. Its action is much more rapid and complete, and generally more persistent. I have almost always seen from ten to twenty inspirations suffice, sometimes fewer. Hence the time of the surgeon is saved; and that preliminary state of excitement which pertains to all narcotizing agents being curtailed, or, indeed, practically abolished, the patient has not the same degree of tendency to exhilaration and talking.

3. Most of those who know from previous experience the sensations produced by ether inhalation, and who have subsequently breathed the chloroform, have strongly declared the inhalation and influence of chloroform to be far more agreeable and pleasant than those of ether.

4. I believe that, considering the small quantity requisite as compared with ether, the use of chloroform will be less expensive than that of ether, more especially as there is every prospect that the means of forming it may be simplified and cheapened.

5. Its perfume is not unpleasant, but the reverse; and the odour of it does not remain for any length of time attached to the clothes of the attendant, or exhaling in a disagreeable form the lungs of the patient, as so generally happens with sulphuric ether.

6. Being required in much less quantity, it is much more portable and transmissible than sulphuric ether.

7. No special kind of inhaler or instrument is necessary for its exhibition. A little of the liquid diffused upon the interior of a hollow-shaped sponge, or a pocket handkerchief, or a piece of linen or paper, and held over the mouth and nostrils so as to be fully inhaled, generally suffices in about a minute or two to produce the desired effect.

I have had an opportunity of using chloroform with perfect success in

*In making a variety of experiments upon the inhalation of different volatile chemical liquids, I have, in addition to perchloride of formyle, breathed chloride of hydro-carbon, acetone, nitrate of oxide of ethyle, benzin, the vapour of iodoform, &c. I may probably take another opportunity of describing the results. It is, perhaps, worthy of remark, that, in performing his experiments upon inhalation, Sir Humphrey Davy confined his attention to the inspiration of gases, and does not seem to have breathed any volatile liquid.

several surgical operations (removal of tumours, of necrosed bone, partial amputation of the great toe,) and in tooth-drawing,* opening abscesses, for annulling the pain of dysmenorrhœa and of neuralgia; in two or three cases where I was using deep and otherwise very painful galvano-puncture for the treatment of ovarian dropsy; in removing a very large fibrous tumour from the posterior wall of the uterus by enucleation, &c.†

I have employed it also in obstetric practice with entire success. The lady to whom it was first exhibited during parturition had been previously delivered in the country by craniotomy, after a labour of three days' duration. In this, her second confinement, pains supervened a fortnight before the full time. Three hours and a half afrer they commenced, and ere the dilatation of the os uteri was completed, I placed her under the influence of the chloroform, by moistening, with half a teaspoonful of the liquid, a pocket-handkerchief rolled up in a funnel shape, and with the broad or open end of the funnel placed over her mouth and nostrils. In consequence of the evaporation of the fluid, it was once more renewed in about ten or twelve minutes. The child was expelled in about twenty-five minutes after the inhalation was begun. The mother subsequently remained longer soporose than commonly happens after ether. The squalling of the child did not, as usual, rouse her; and some minutes elapsed after the placenta was expelled, and after the child was removed by the nurse into another room, before the patient awoke. She then turned round, and observed to me that she had "enjoyed a very comfortable sleep, and, indeed, required it, as she was so tired,‡ but would now be more able for the work before her." I evaded entering into conversation with her, believing, as I do, that the most complete possible quietude forms one of the principal secrets for the successful employment of either ether or chloroform. In a little time she again remarked that she was afraid her "sleep had stopped the pains." Shortly afterwards her infant was brought in by the nurse from the adjoining room, and it was a matter of no small difficulty to convince the astonished mother that the labour was entirely over, and that the child presented to her was really her "own living baby."

* A young dentist, who has himself had two teeth extracted lately—one under the influence of ether, and the other under the influence of chloroform—writes me the following statement of the results:—"About six months ago I had an upper molar tooth extracted whilst under the influence of ether, by Mr. Imlach. The inhalation was continued for several minutes before I presented the usual appearance of complete etherization; the tooth was extracted, and, although I did not feel the least pain, yet I was conscious of the operation being performed, and was quite aware when the crash took place. Some days ago I required another molar extracted on account of toothache, and this operation was again performed by the same gentleman. I inhaled the vapour of chloroform, half a drachm being poured upon a handkerchief for that purpose, and held to my nose and mouth. Insensibility took place in a few seconds; but I was so completely *dead* this time that I was not in the very slightest degree aware of anything that took place. The subsequent stupifying effects of chloroform went off more rapidly than those of ether, and I was perfectly well and able for my work in a few minutes."

† I have now exhibited chloroform to about fifty individuals, and in not one has the slightest bad effect of any kind resulted.

‡ In consequence of extreme anxiety at the unfortunate result of her previous confinement, she had slept little for none or one or two nights preceding the commencement of her present accouchement.

Perhaps I may be excused from adding that, since publishing on the subject of ether inhalation in midwifery, seven or eight months ago, and then for the first time directing the attention of the profession to its great use and importance in natural and morbid parturition, I have employed it, with few and rare exceptions, in every case of labour that I have attended, and with the most delightful results; and I have no doubt whatever that some years hence the practice will be general. Obstetricians may oppose it, but I believe our patients themselves will force the use of it upon the profession.* I have never had the pleasure of watching over a series of better and more rapid recoveries; nor once witnessed any disagreeable result follow either to mother or child; whilst I have now seen an immense amount of maternal pain and agony saved by its employment. And I most conscientiously believe that the proud mission of the physician is distinctly twofold, namely, to alleviate human suffering, as well as to preserve human life.

In some remarks which I published in "The Monthly Journal of Medical Science" for Sept., p. 154, relative to the conditions for ensuring successful etherization in surgery, I took occasion to insist upon the three following leading points:—"First, the patient ought to be left, as far as possible, in a state of absolute quietude and freedom from mental excitement, both during the induction of etherization, and during his recovery from it. All talking and all questioning should be strictly prohibited. In this way any tendency to excitement is eschewed, and the proper effect of the ether inhalation more speedily and certainly induced. And secondly, with the same view, the primary state of exhilaration should be entirely avoided, or at least reduced to the shortest possible limit, by impregnating the respired air as fully with the ether-vapour as the patient can bear, and by allowing it to pass into the lungs, both by the mouth and nostrils, so as rapidly and at once to induce its complete and anæsthetic effect * * * a very common, but certainly a very unpardonable error, being to exhibit an imperfect and exciting, instead of a perfect and narcotizing, dose of the vapour. Many of the alleged failures and misadventures are doubtless entirely attributable to the neglect of this simple rule; not the principle of etherization, but the mode of putting it in practice being altogether to blame. But, thirdly, whatever means or mode of etherization is adopted, the most important of the conditions required for procuring a satisfactory and successful result from its employment in surgery, consists in obstinately determining to avoid the commencement of the operation itself, and never venturing to apply the knife, until the patient is under the full influence of the ether-vapour, and thoroughly and indubitably soporized by it."

In fulfilling all these indications, the employment of chloroform evidently offers great and decided advantages in rapidity, facility, and efficiency over the employment of ether. When used for surgical purposes, I would advise it to be given upon a handkerchief, gathered up

*I am told that the London physicians, with two or three exceptions only, have never yet employed ether inhalation in midwifery practice. Three weeks ago I was informed in a letter from Professor Montgomery, of Dublin, that he believed that in that city, up to that date, it had not been used in a single case of labour.

into a cup-like form, in the hand of the exhibitor, and the open end of the cup placed over the mouth and nostrils of the patient. For the first inspiration or two it should be held at the distance of an inch or so from the face; and then more and more closely applied to it. To ensure a full and perfect anæsthetic effect—more especially when the operation is to be severe—a teaspoonful of the chloroform should at once be placed upon the hollow of the handkerchief, and immediately held to the face of the patient. Generally a snoring sleep very speedily supervenes; and when it does it is a perfect test of the superinduction of complete insensibility. But many patients are quite anæsthetic without this symptom.

As an illustration of the influence of this new anæsthetic agent, I will select and append notes of two operations performed with it on Friday last by Professor Miller—the first in the Royal Infirmary of Edinburgh,* the other in private practice. The notes and remarks are in Mr. Miller's own words.

Case I.—"A boy, four or five years old, with necrosis of one of the bones of the forearm. Could speak nothing but Gælic. No means, consequently, of explaining to him what he was required to do. On holding a handkerchief, on which some chloroform had been sprinkled, to his face, he became frightened, and wrestled to be away. He was held gently, however, by Dr. Simpson, and obliged to inhale. After a few inspirations he ceased to cry or move, and fell into a sound snoring sleep. A deep incision was now made down to the diseased bone; and, by the use of the forceps, nearly the whole of the radius, in the state of sequestrum, was extracted. During this operation, and the subsequent examination of the wound by the finger, not the slightest evidence of the suffering of pain was given. He still slept on soundly, and was carried back to his ward in that state. Half an hour afterwards he was found in bed, like a child newly awakened from a refreshing sleep, with a clear, merry eye, and placid expression of countenance, wholly unlike what is found to obtain after ordinary etherization. On being questioned by a Gælic interpreter who was found among the students, he stated that he had never felt any pain, and that he felt none now. On being shown his wounded arm, he looked much surprised, but neither cried nor otherwise expressed the slightest alarm."

Case II.—"A young lady wished to have a tumour (encysted) dissected out from beneath the angle of the jaw. The chloroform was used in small quantity, sprinkled upon a common operation sponge. In considerably less than a minute she was sound asleep, sitting easily in a chair, with her eyes shut, and with her ordinary expression of countenance. The tumour was extirpated, and a stitch inserted, without any pain having been either shown or felt. Her sensations throughout, as she subsequently stated, had been of the most pleasing nature; and her manageableness during the operation was as perfect as if she had been a wax doll or a lay figure.

* Professor Dumas, (of Paris,) Mr. Milne Edwards, Dr. Christison, Sir George Ballingall, and a large collection of professional gentlemen and students witnessed this operation and two others performed with similar success by Professor Miller and Dr. Duncan.

"No sickness, vomiting, headache, salivation, uneasiness of chest, in any of the cases. Once or twice a tickling cough took place in the first breathings."—*London Medical Times.*

